# VirPhyKit: An Integrated Toolkit for Viral Phylogeographic Analysis

**DOI:** 10.1002/ece3.72796

**Published:** 2025-12-17

**Authors:** Yuqi Yin, Jianguo Shen, Zhenguo Du, Simon Y. W. Ho, Fangluan Gao

**Affiliations:** ^1^ State Key Laboratory of Agricultural and Forestry Biosecurity Fujian Agriculture and Forestry University Fuzhou China; ^2^ Fujian Key Laboratory for Technology Research of Inspection and Quarantine, Technology Center of Fuzhou Customs District Fuzhou China; ^3^ School of Life and Environmental Sciences University of Sydney Sydney New South Wales Australia

**Keywords:** Bayesian phylogeography, data subsampling, molecular dating, region‐randomization test, spatial diffusion

## Abstract

The evolutionary, demographic, and phylogeographic histories of viral pathogens can be inferred from their genomic sequence data. Bayesian phylogeographic approaches, for example, can be used to reconstruct the spatial dynamics of pathogen transmission. Although a range of tools is available for evolutionary analyses of viral pathogens, these typically focus on specific stages of the workflow, leaving a need for an integrated framework. We introduce VirPhyKit, a toolkit that integrates 12 specialized modules through an intuitive point‐and‐click interface. VirPhyKit streamlines key tasks in phylogeographic analysis, including sequence curation, spatiotemporal subsampling, migration pattern analysis, and molecular dating. Furthermore, it offers built‐in functionality to generate high‐quality figures that are suitable for publication. As a stand‐alone application supported on Windows and Linux operating systems, VirPhyKit promises to be a valuable resource for researchers studying viral ecology and evolution. The software and source code are available at https://github.com/BioEasy/VirPhyKit.

## Graphical abstract

A new toolkit for phylogeographic analysis called VirPhyKit is developed. The toolkit integrates 12 specialized modules within an intuitive point‐and‐click interface, streamlining key tasks such as sequence curation, spatiotemporal subsampling, migration pattern analysis, and molecular dating.

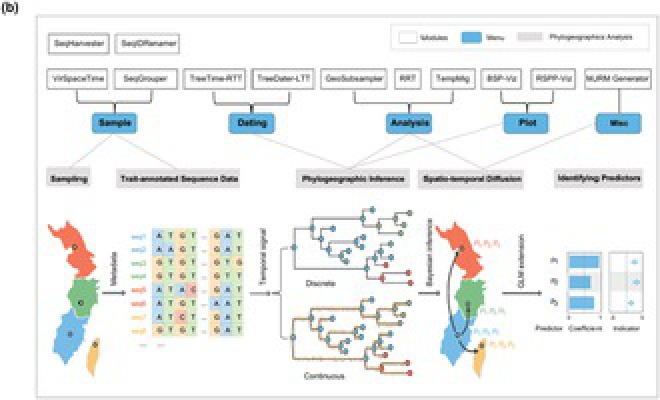

## 1 Introduction

Phylogeographic and molecular dating approaches have become indispensable tools in studies of viral pathogens and molecular epidemiology, enabling researchers to trace the spread of diseases across clinical, environmental, and agricultural contexts (Faria et al. 2011). These methods are able to reconstruct transmission pathways by analyzing phylogenetic trees that have been annotated with either discrete or continuous ancestral character states, typically representing the geographic sampling locations. Viral phylogeographic analyses are most often performed in a Bayesian statistical framework, with the BEAST software suites (versions 1.x and 2.x) being the most widely used platforms (Baele et al. 2025; Bouckaert et al. 2019).

Bayesian phylogeographic studies integrate genetic sequences, temporal information, and spatial data into comprehensive phylodynamic models. The power of these methods has grown considerably with the increasing availability of genetic data from high‐throughput sequencing technologies (e.g., Biek et al. 2015). However, analyzing these data sets presents substantial challenges, including time‐intensive steps such as data retrieval and management, curation of GenBank entries, metadata integration, and subsampling strategies. Furthermore, the inferred temporal migration patterns and ancestral states need to be visualized in an informative and appealing way so that the results can be presented effectively.

**FIGURE 1:**
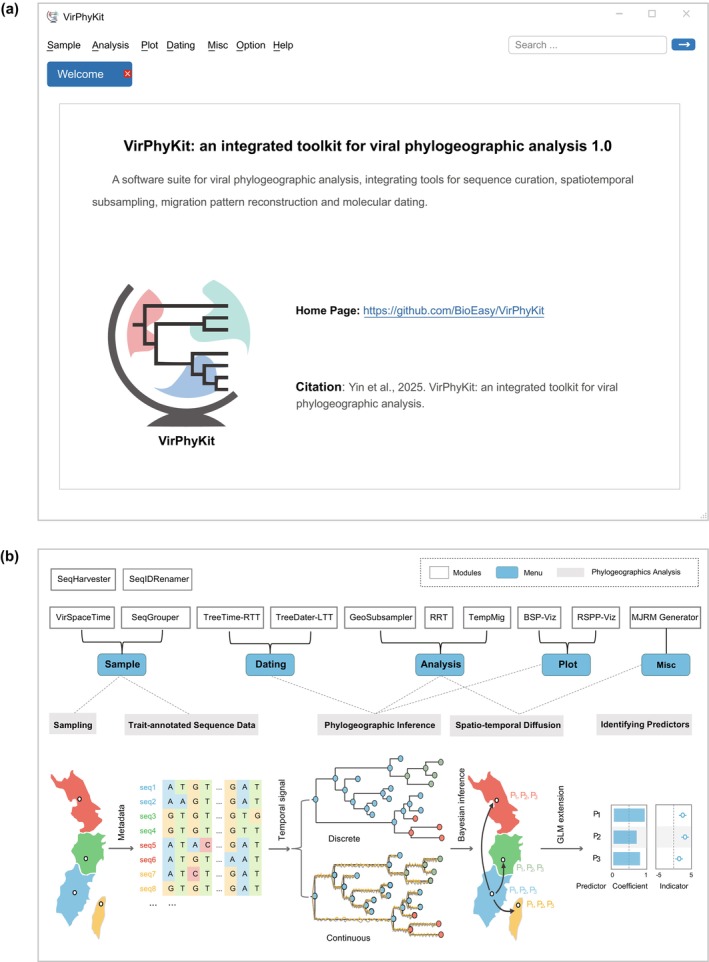
Overview of the VirPhyKit interface and its modules. (a) Module controls are organized into five toolboxes: “Sample” for integrating sequence metadata; “Analysis” for subsampling, region randomization, and temporal migration analyses; “Dating” for molecular dating; “Plot” for visualizing phylogeographic inference results; and “Misc” for generating Markov jump and reward matrices. (b) A schematic flowchart illustrating the functionality of each VirPhyKit module, highlighting the specific tasks they support within the phylogeographic analysis pipeline.

Several tools have been developed for phylogeographic analysis, but most of these have been designed for specific components of the workflow (Table 1). For instance, the web application EvoLaps specializes in visualizing phylogeographic patterns across time and continuous space (Chevenet et al. 2021), whereas PhyCova focuses exclusively on identifying covariates associated with pathogen dispersal (Blokker et al. 2022). To provide a more integrated environment for viral phylogeographic analysis, we present a comprehensive toolkit called VirPhyKit (short for “Viral Phylogeographic Analysis Toolkit”). This toolkit incorporates 12 specialized modules accessible through an interactive point‐and‐click interface, enabling sequence curation, spatiotemporal subsampling, migration pattern analysis, and molecular dating (Figure 1; Table 2). In addition, VirPhyKit includes integrated functionality to generate publication‐quality figures, enabling the efficient creation of high‐quality visualizations for communicating the outcomes of phylogeographic analyses.

**TABLE 1:** Comparison of features in VirPhyKit and other tools.

Key features	SpreaD3^a^	TempEst^b^	IcyTree^c^	Treedater^d^	TreeTime^e^	Nextstrain^f^	PhyloSuite^g^	EvoLaps^h^	VirPhyKit
Metadata integration and visualization									
Sequence fetch and download	×	×	×	×	×	√	√	×	√
Sequence renaming, grouping, and visualization	×	×	×	×	×	×	×	×	√
Mapping the location and timing of isolates	×	×	×	×	×	×	×	×	√
Phylogeographic analyses					√^1^	√^1^			
Dating and temporal signal analysis	×	√	×	√	√	√	×	×	√
Subsampling analysis	×	×	×	×	×	√	×	×	√
Region‐randomization test	×	×	×	×	×	×	×	×	√
Migration‐over‐time analysis	×	×	×	×	×	×	×	×	√
Lineage‐through‐time analysis	×	×	×	√	×	×	×	×	√
Phylogeographic output visualization	√^1^					√^1^		√^1^	
Bayesian skyline plot visualization	×	×	×	×	×	×	×	×	√
Root state posterior probability visualization	×	×	√	×	×	√	×	×	√

^1^ Additional analysis and visualization features available.

^a^ Bielejec et al. (2011).

^b^ Rambaut et al. (2016).

^c^ Vaughan (2017).

^d^ Volz and Frost (2017).

^e^ Sagulenko et al. (2018).

^f^ Hadfield et al. (2018).

^g^ Zhang et al. (2020).

^h^ Chevenet et al. (2021).

**TABLE 2:** Overview of the 12 modules in VirPhyKit.

Menu	Module	Brief description
Sample	SeqHarvester (Sequence Metadata Harvester)	Retrieves viral isolates from GenBank, displays comprehensive metadata for filtering and download
	SeqIDRenamer (Sequence Identifier Renamer)	Provides a simple workflow for batch processing of sequence IDs using custom naming rules
	SeqGrouper (Sequence Grouping Tool)	Groups viral sequences into customizable categories (e.g., geographic region, host, or user‐defined criteria), with automated distribution reports for research and surveillance
	VirSpaceTime (Viral Space–Time Visualizer)	Visualizes spatial and/or temporal distributions of viral isolates
Analysis	RRT (Region‐Randomization Test)	Detects sampling bias in phylogeographic analyses using a statistical approach
	TempMig (Temporal Migration Tracker)	Reconstructs and visualizes spatial diffusion of viral pathogens through time
	GeoSubsampler (Sequence Geographic Subsampler)	Generates balanced sequence subsets by either geographic region or sample size, with integrated support for bootstrap analysis
Plot	BSP‐Viz (Bayesian Skyline Plot Visualizer)	Visualizes the export results of Bayesian skyline plots
	RSPP‐Viz (Root State Posterior Probability Visualizer)	Visualizes root state posterior probabilities inferred from trait‐annotated MCC trees or MultiTypeTree
Dating	TreeTime‐RTT (Root‐to‐tip Regression by TreeTime)	Assesses clock‐like evolutionary patterns through linear regression of root‐to‐tip distances against sampling dates in TreeTime
	TreeDater‐LTT (Lineage‐Through‐Time with Treedater)	Conducts a lineage‐through‐time (LTT) analysis using Treedater
Misc	MJRM (Markov Jumps and Rewards Matrix Generator)	Automatically constructs Markov jump and reward matrices from user‐defined discrete traits (e.g., geographic location or host species) for phylogeographic analysis

## 2 Implementation

### 2.1 Sequence Curation and Metadata Organization

Genomic sequence data are fundamental for studying the evolution, transmission, and epidemiology of viral pathogens (Geoghegan and Holmes 2018; Hill et al. 2021). Public databases, such as NCBI's GenBank, serve as the primary sources of viral genomic sequences, yet the rapid growth in sequencing efforts has resulted in datasets that are increasingly large, heterogeneous, and inconsistently annotated. Existing sequence management tools are not designed to handle this complexity, because they are often distributed across different platforms and offer only narrow, task‐specific functions. As a result, retrieving, organizing, and processing viral sequences, particularly at scale, remains a major bottleneck in phylogeographic and evolutionary research. VirPhyKit addresses these issues by providing integrated functions for downloading, renaming, organizing, and grouping sequence information, as well as visualizing sampling data. VirPhyKit implements a suite of 12 tools: SeqHarvester, SeqIDRenamer, SeqGrouper, VirSpaceTime, GeoSubsampler, RRT, TempMig, TreeTime‐RTT, TreeDater‐LTT, BSP‐Viz, RSPP‐Viz, and MJRM Generator.

The goal of SeqHarvester, SeqIDRenamer, and SeqGrouper is to streamline the processing of viral sequences and their associated metadata. SeqHarvester is a retrieval tool for viral sequence data, enabling users to fetch isolates from GenBank using either virus names or accession numbers. The utility displays comprehensive metadata for all matching records, allows sequence‐type filtering, and downloads the selected data to user‐specified directories. The SeqIDRenamer tool is designed for the rapid renaming of sequence identifiers in FASTA files. It provides a straightforward workflow for batch processing sequence IDs according to user‐defined naming rules. SeqGrouper is a utility that groups viral sequences into customizable categories (e.g., geographic region, host, or user‐defined criteria) and generates automated distribution reports to support research and surveillance efforts.

In Bayesian phylogeographic studies of samples with discrete location states, researchers face the nontrivial task of defining geographic sampling locations. This often involves either grouping multiple sites into a single discrete state or partitioning a continuous geographic or administrative region into multiple distinct states (Lemey et al. 2009). Mapping the collection sites of viral isolates can provide an intuitive visual reference to support decisions about the merging or partitioning of regions. Additionally, phylogeographic studies benefit greatly from an estimate of the timescale of spatial diffusion. The timescale can be inferred using tip‐dating, a technique that jointly estimates the substitution rate and timing of evolutionary divergences using time‐stamped sequences (Rieux and Balloux 2016). However, this method requires that the data set has sufficient temporal signal, whereby the sampling period is wide enough to capture an adequate amount of evolutionary change (Drummond et al. 2003). To facilitate the visualization of spatiotemporal distribution patterns among viral isolates, we developed the VirSpaceTime utility. This tool enables users to explore the spatial distribution of viral isolates, thereby informing decisions about geographic grouping or subdivision. Furthermore, the tool allows users to visualize the temporal distribution of sample sizes across different geographic locations, providing a clearer understanding of the sampling window and its implications for molecular dating analysis.

### 2.2 Mitigation of Sampling Bias

In viral phylogeography, models of spatiotemporal evolution are highly susceptible to sampling bias. Non‐uniform sampling across space and time can distort any inferences made from the sequence data (Guindon and De Maio 2021). For instance, underrepresentation of certain regions can obscure the true spatiotemporal dynamics, while oversampling can artificially inflate inferred migration rates (Baele et al. 2017). These biases are particularly problematic in ancestral state reconstructions that employ Bayesian stochastic search variable selection to identify the most plausible pathways of phylogeographic diffusion (e.g., Lemey et al. 2009).

To enable researchers to evaluate and mitigate sampling biases in viral sequence data, we developed two modules in VirPhyKit: GeoSubsampler and RRT (region‐randomization test). GeoSubsampler is a specialized tool that aims to correct sampling deviations by performing region‐stratified subsampling of FASTA‐formatted sequence data sets. It produces balanced subsets of sequences based on geographic region or sample size and includes integrated support for bootstrap analysis. RRT is a statistical method designed to detect and correct for sampling bias in phylogeographic analysis. It compares ancestral root state probabilities between the original data set and replicate data sets with permuted location labels, thereby providing a randomization test that can account for the biasing effect of oversampled regions.

### 2.3 Analysis and Visualization of Viral Diffusion Over Time

To study the spatial diffusion patterns of viral pathogens over time, a reference script by Brynildsrud et al. (2018) is commonly used to analyze the load and direction of the inferred maximum‐clade‐credibility (MCC) trees (e.g., Wu et al. 2022; Xu et al. 2021). Although metadata from nodes and tips can be extracted to reconstruct the diffusion patterns of viruses through time, the complexity of this analytical process has remained challenging for users. To address this gap, we offer the TempMig and TempMigPlotter utilities within VirPhyKit. These modules process either the trait‐annotated MCC tree or the output tree of a structured coalescent analysis (or its approximations) to generate time‐resolved migration matrices and interactive visualizations.

### 2.4 Root‐to‐Tip Regression and Lineage‐Through‐Time Analyses

Tip‐dating relies on a sufficient temporal signal to yield reliable inferences. Therefore, a critical preliminary analysis involves evaluating the strength of the signal. This can be done using methods such as the date‐randomization test (Duchene et al. 2015) or Bayesian model selection (Duchene et al. 2020), but temporal signal is more commonly evaluated using root‐to‐tip regression (RTT) analysis in tools such as TempEst (Rambaut et al. 2016) and TreeTime (Sagulenko et al. 2018). However, these RTT regression tools lack the capability to explicitly examine how geographic groupings structure the scatterplot. To overcome this limitation, VirPhyKit includes an extension named TreeTime‐RTT, which visualizes the temporal signal stratified by geographic partitions (or other customizable categories). This offers deeper insights into the interactions between sampling time, substitution rate, and spatial structure.

By enabling a rapid evaluation of temporal signal, RTT regression serves as a useful qualitative complement to other formal methods. However, it assumes a strict molecular clock and cannot satisfactorily accommodate rate variation across lineages (Firth et al. 2010). To handle data with appreciable levels of among‐lineage rate variation, we integrate TreeDater‐LTT into VirPhyKit. This module is a wrapper of TreeDater, an extensible relaxed‐clock method designed specifically to analyze rapidly evolving viral lineages (Volz and Frost 2017). In addition to conducting a lineage‐through‐time (LTT) analysis, TreeDater‐LTT retains the core functionalities of its parent software, including inference of evolutionary rates and the time to the most recent common ancestor.

### 2.5 Visualization of Demographic History and Root State Probabilities

The Bayesian skyline plot (BSP) is one of the most widely used demographic models, allowing the effective population size to change in a piecewise fashion at coalescent events (Drummond et al. 2005). We developed BSP‐Viz to allow the visualization of viral population dynamics inferred using the BSP in the Bayesian phylogenetic software BEAST. This tool supports both single‐file and batch processing modes.

Summarizing and visualizing root state posterior probabilities (RSPP) inferred from MCC trees annotated with discrete traits or structured coalescent analyses (including approximations) can be challenging for new users (Lemey et al. 2009). Performing this task using a text editor is not only difficult but also prone to error. VirPhyKit addresses this issue with RSPP‐Viz, which enables users to visualize and interact with RSPP results intuitively and efficiently.

The MJRM Generator utility in VirPhyKit automatically constructs Markov jump and reward matrices from user‐defined discrete traits (e.g., geographic location or host species) for phylogeographic analysis. These matrices are then seamlessly integrated into BEAST‐compatible XML configuration files.

## 3 Worked Example

To demonstrate the core functional modules of VirPhyKit, we use data sets from our previous phylogenetic studies of tomato mosaic virus (Xu et al. 2021), rice stripe virus (Wu et al. 2022), and potato virus S (Duan et al. 2018). These data sets are provided alongside the toolkit. Our earlier phylogeographic investigations of these viruses offered valuable insights into their spread and demographic histories, making them well‐suited for illustrating the capabilities of VirPhyKit.

Before using VirPhyKit, users need to configure the installation paths for R, Python, and Perl via “Options ➔ Environment Settings”. After clicking the “Check” button, the toolkit automatically verifies all required Python and R package dependencies, identifies any that are missing, and offers to install them.

### 3.1 Retrieving Viral Sequences and Metadata

The first step in any phylogeographic analysis is to obtain high‐quality sequence data. Using the SeqHarvester utility in VirPhyKit, users can retrieve sequences from GenBank by either providing a full virus name or uploading a text file of accession numbers. Before starting, a local working directory must be specified. When querying by virus name, the utility retrieves and displays complete metadata for all available GenBank isolates in the “Results” panel, allowing users to select specific genomic segments through the “Genome Segments” dropdown menu. Ultimately, SeqHarvester outputs FASTA sequences and a consolidated list of accessions, while simultaneously maintaining real‐time progress and error logs to ensure date integrity.

Following sequence retrieval, the SeqGrouper utility facilitates the extraction and organization of critical metadata, such as sampling dates for tip‐dating and sampling locations or hosts for coding discrete traits. The process begins with the upload of input data either as a text file containing accession numbers or as a GenBank file (.gb). The user must then check the “Enable” option before selecting either the default grouping rules, which organize data by region, or uploading a custom grouping table. Clicking “Show Table” populates the interface with the viral isolate information, at which point the user selects the desired columns for grouping and clicks the “View” button to observe the group distribution. The utility automatically appends these newly grouped categories as additional columns to the data table for subsequent phylogeographic grouping.

To aid in data verification, SeqGrouper includes a preview function that visualizes group distributions via pie charts and presents the results in an editable table. The use of a mapping table can be toggled with a checkbox; if left disabled, a default mapping is applied. All results are exported as CSV files. Furthermore, the associated viral isolate metadata, including collection date, geographic origin, and host species, can be generated as a supplementary table to directly support downstream phylogeographic analysis.

### 3.2 Visualizing Spatial and Temporal Distributions of Viral Isolates

**FIGURE 2:**
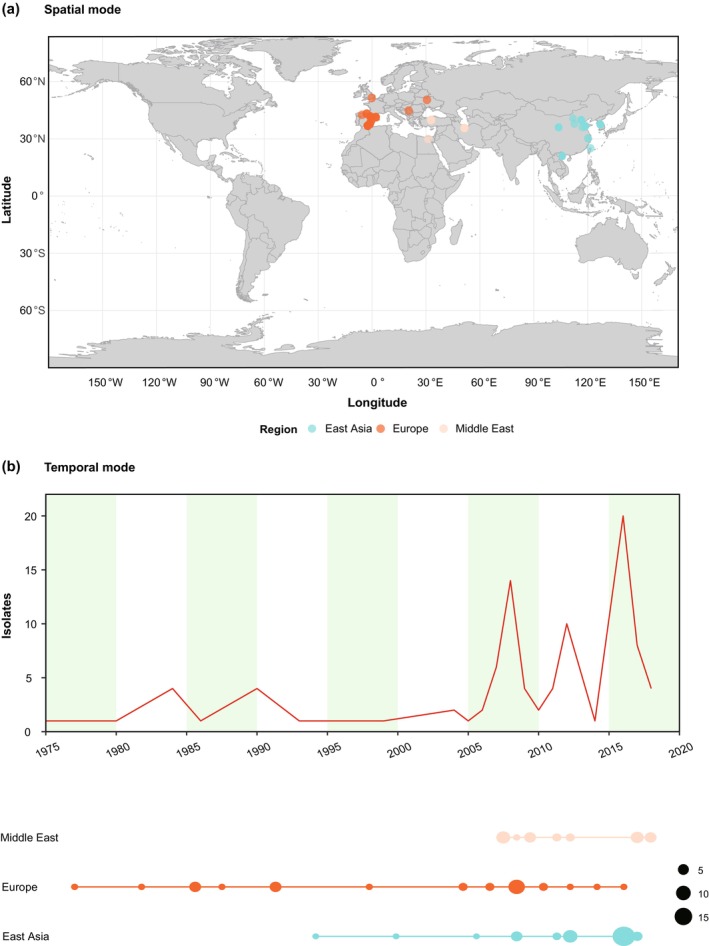
Spatial and temporal distribution of viral isolates of tomato mosaic virus generated using the VirSpacTime utility. (a) Geographic map showing the collection sites of the isolates (spatial mode); (b) Temporal distribution of the sample size across the different geographic locations (temporal mode).

To visualize spatial or temporal distribution patterns among viral isolates, the user first selects the desired mode (temporal or spatial distribution). A tab‐delimited text file is then uploaded, containing either geographic coordinates for spatial analysis or population sample sizes over time for temporal analysis. After the output directory and filename are specified, clicking the “Generate” button produces the visualization and exports the results as a PDF document. This provides researchers with an intuitive overview of the geographic distribution and temporal sampling window of the dataset. As a practical demonstration, Figure 2 illustrates the spatial and temporal distribution patterns of tomato mosaic virus isolates generated using the VirSpaceTime utility.

### 3.3 Subsampling Analysis and Region‐Randomization Test

In VirPhyKit, the GeoSubSampler tool provides two subsampling modes: normal and bootstrap. In normal mode, the user first selects an output directory and then specifies the number of sequences to be randomly subsampled, either from the entire data set or from a user‐defined geographic region. The tool subsequently extracts the specified number of sequences randomly from the chosen data set or region. For region‐specific subsampling, all sequences must first be relabeled with their corresponding geographic region identifiers. In bootstrap mode, a bootstrapping approach described by Gao et al. (2017) is activated to standardize the sample size according to the smallest geographic region. To perform the analysis, the user switches to bootstrap mode, specifies a local folder (as in normal mode), and defines the number of replicates. The resulting subsampled data sets in FASTA format are saved in the designated output directory. Detailed logs, including the number of sequences retained and their geographic distribution, are displayed in the user interface.

**FIGURE 3:**
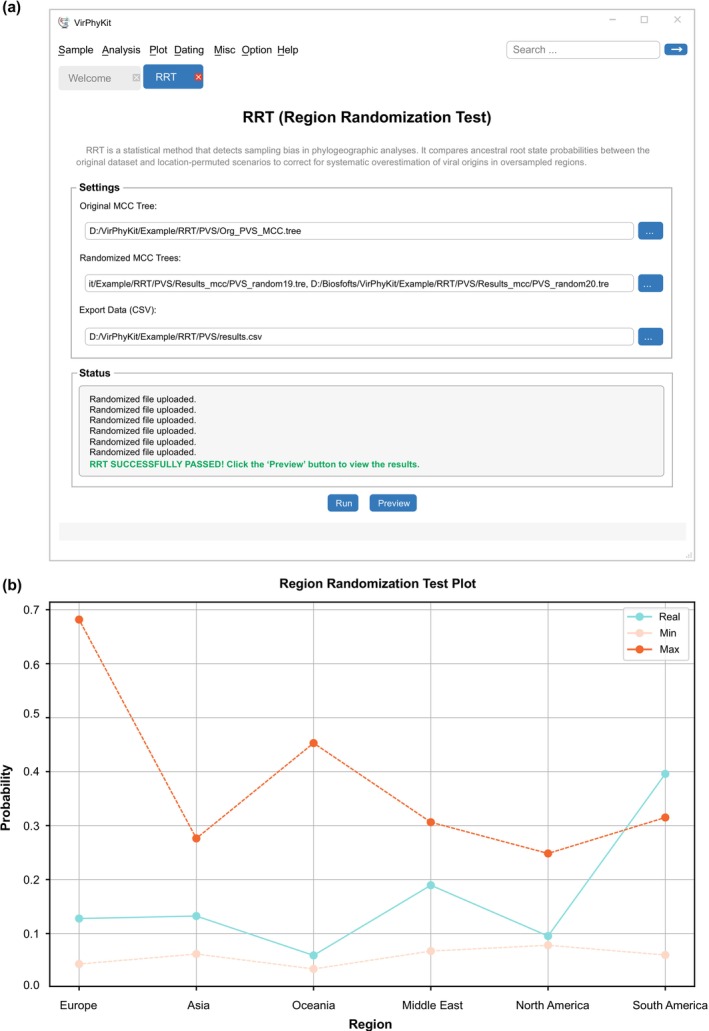
Analysis and visualization of the region‐randomization test (RRT) of potato virus S performed with VirPhyKit. (a) Screenshot of the user interface for conducting a RRT; (b) Results of RRT. The posterior probabilities of root regions are shown on the *y*‐axis. The dashed lines represent the maximum and minimum posterior probability values estimated from 20 region‐randomized replicates. The solid blue line indicates the estimate from the original data set.

To perform a region‐randomization test and assess potential sampling bias in a data set, the user must load the original MCC tree and multiple region‐randomized trees. The tool parses geographic regions (e.g., countries or continents) and their corresponding posterior probabilities from the original MCC tree and region‐randomized trees and then compiles the results into a summary table. This table includes a “Real” row (posterior probabilities from the original tree), along with “Min” and “Max” rows representing the probability ranges observed across region‐randomized replicates. The absence of significant bias is inferred when the maximum posterior probability from the original tree falls outside the range observed across the region‐randomized replicates. Results are exported in CSV format, and the tool includes a visualization module that generates line plots comparing the Real, Min, and Max values. In the example data set (Duan et al. 2018), Bayesian analysis placed the root of the MCC tree in South America with a posterior probability of 0.4. This value falls outside the probability range obtained from analyses of 20 region‐randomized data sets, so we conclude that the result is reliable (Figure 3).

### 3.4 Investigating Temporal Migration Patterns

To investigate the patterns of spatial diffusion over time, the initial step requires generating the migration matrix from either the trait‐annotated MCC tree or the output tree of a structured coalescent analysis (or its approximations). This workflow comprises internal node labeling, conversion to standardized Newick format, and systematic extraction of node attributes (including node ID, height, branch length, and geographic state). Using these data, the TempMig utility constructs a migration matrix that quantifies annual migration events between and within geographic locations.

**FIGURE 4:**
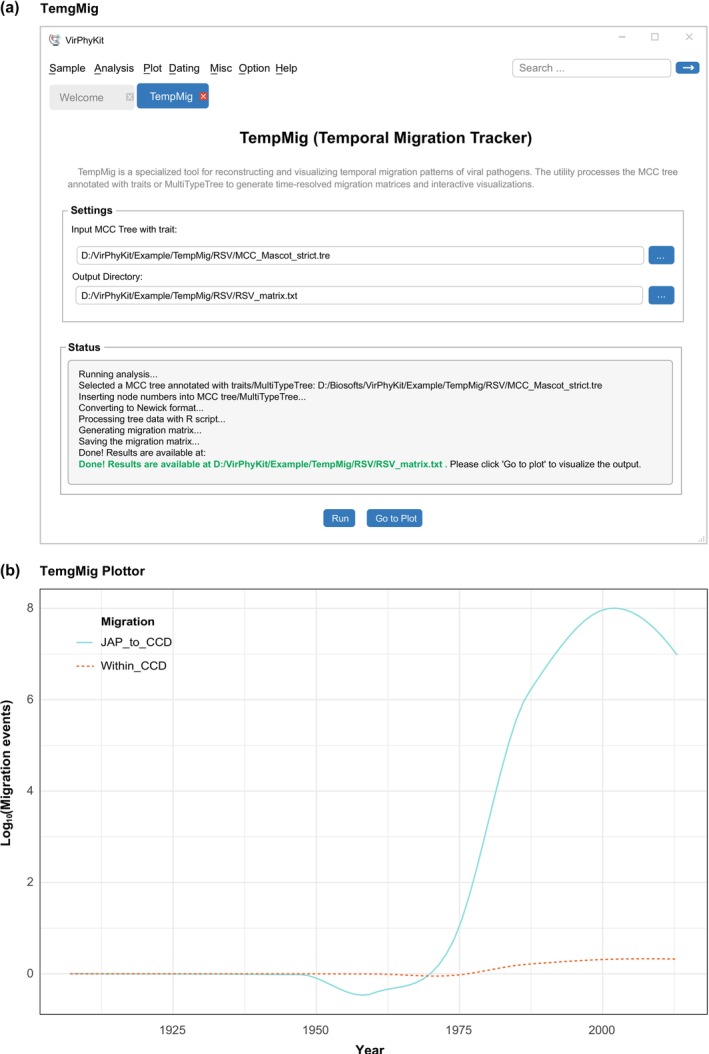
Analysis and visualization of temporal migration pathways of rice stripe virus within the central China double cropping region. (a) The analysis was conducted using the TempMig utility, and (b) the results were visualized with the TempMigPlotter utility, both part of the VirPhyKit toolkit.

Following matrix generation, the TempMig Plotter visualizes migration dynamics through time. Users can load migration matrices via the “Go to Plot” button, select migration directions through an interactive checkbox table (e.g., A_to_B for inter‐location migration or A_to_A for intra‐location migration), and specify an output directory for PDF exports. Two visualization modes are supported: Normal, which uses line plots of raw migration counts, and Smooth, which employs smoothed curves that emphasize temporal trends. The outputs of both modes are presented in Figure 4, matching the results of Wu et al. (2022).

### 3.5 Visualizing BSP and ASR Outputs

The BSP‐Viz utility in VirPhyKit enables the visualization of effective population size (*N*_e_) through time, based on the output of BSP analysis using BEAST (.tsv file). The user selects a .tsv file containing time, median, and 95% credibility interval data, specifies an output PDF path, and chooses the time axis orientation (forward or reverse), with an optional auxiliary axis for enhanced clarity. The tool generates skyline plots displaying median *N*_e_ on a logarithmic scale with shaded 95% credibility intervals. Batch processing supports simultaneous analysis of multiple .tsv files, and visualization parameters can be customized through the “Hue Harmony” interface.

The RSPP‐Viz utility visualizes posterior probabilities of ancestral geographic states at the root of trait‐annotated MCC trees. The user provides a tree file and an output directory, after which the tool automatically extracts trait information and posterior probabilities using regular expressions. Two visualization styles are supported: pie charts illustrate proportional probabilities with percentage annotations, as demonstrated by Long et al. (2024); while histograms display probability distributions with numerical labels in the manner of Duan et al. (2018). The tool supports batch processing, with output PDFs automatically named according to their corresponding input files. Color schemes can be customized via the “Hue Harmony” interface.

### 3.6 Root‐to‐Tip Regression and Lineage‐Through‐Time Analyses

**FIGURE 5:**
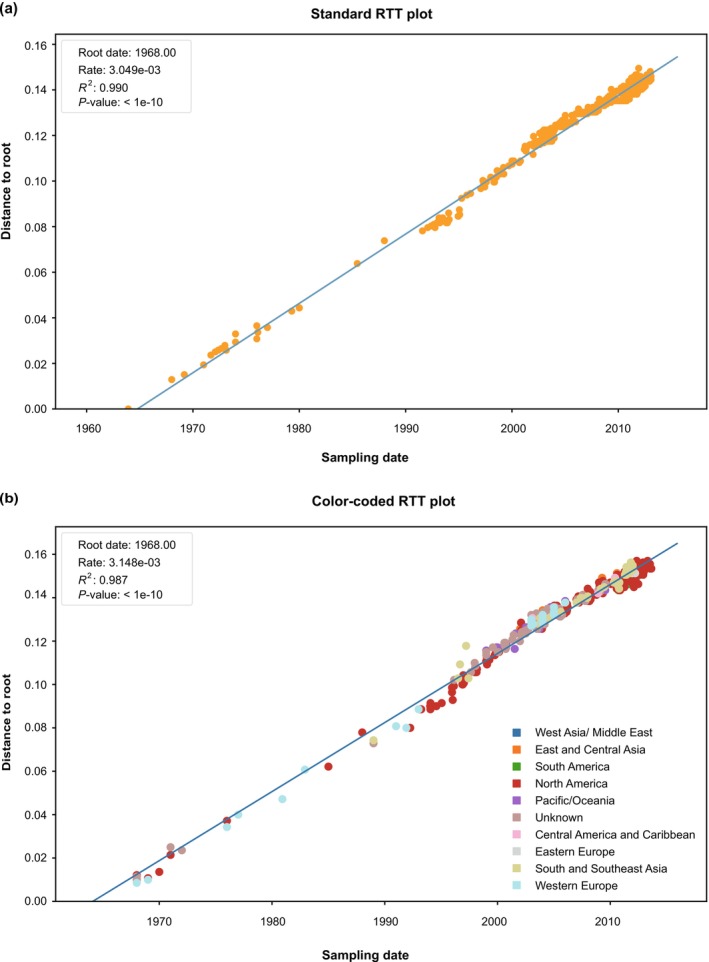
Root‐to‐tip (RTT) regression analysis of influenza A/H3N2 HA sequences. The regression of genetic distances against sampling dates was performed using the TreeTime‐RTT utility to assess the temporal signal. (a) Standard RTT plot with isolates uniformly colored, independent of geographic region or other traits; (b) Color‐coded RTT plot visualizing temporal signal stratified by geographic partitions, where isolates are colored according to their geographic regions.

Before assessing temporal signal in a data set via root‐to‐tip regression analysis, users must first infer a non‐clock phylogenetic tree using a method such as maximum likelihood. The TreeTime‐RTT utility workflow requires three input files: an aligned FASTA sequence file, a Newick‐format tree file, and a CSV metadata file (containing tip names, sampling dates, and optional trait information). A mapping file is optional and only necessary when grouping isolates by traits (e.g., geographic region). After the output directory is specified, clicking the “Run” button initiates the analysis. The outputs include a plot of root‐to‐tip distances against sampling dates (optionally color‐coded by trait), with statistical annotations (*β*, *R* ^2^, and *p*‐value if available), along with a time‐scaled phylogenetic tree illustrating temporal structure (Figure 5a). A legend showing the geographic region groupings is automatically included when a mapping file is enabled (Figure 5b).

To illustrate changes in viral lineage counts over time, the TreeDater‐LTT utility workflow requires a Newick‐format tree file, a metadata CSV file (with tip names and sampling dates), and the sequence length. A lineage‐through‐time plot is optional for users. After the output directory is specified, clicking the “Run” button initiates the analysis. The outputs include molecular clock rates, the time to the most recent common ancestor, and a time‐scaled tree. Results are displayed in the interface log and saved in the specified directory.

## 4 Conclusions

We have developed VirPhyKit, an integrated toolkit designed to streamline the processing of viral phylogeographic analysis by consolidating essential, yet previously fragmented tools. Specifically, this integrated workflow encompasses the acquisition, organization, and preparation of viral sequence data, thereby effectively replacing laborious manual methods. Its core strengths lie in its modular architecture, which integrates 12 specialized modules, an intuitive point‐and‐click interface, and robust cross‐platform compatibility. These features collectively enhance the accessibility and reproducibility of complex spatiotemporal analyses. However, the current version exhibits an inherent trade‐off in flexibility for highly customized analyses when compared with command‐line interfaces. Nevertheless, we anticipate that VirPhyKit will be useful for researchers in viral ecology and evolution. With VirPhyKit being an actively developed project, we welcome bug reports, feedback, and suggestions from the user community. Future efforts will concentrate on optimizing computational efficiency for large datasets and expanding analytical flexibility, ensuring that new features will be continually incorporated to meet emerging research needs.

## Author Contributions

**Yuqi Yin:** software (lead), visualization (lead). **Jianguo Shen:** writing – review and editing (equal). **Zhenguo Du:** writing – review and editing (equal). **Simon Y. W. Ho:** writing – review and editing (equal). **Fangluan Gao:** conceptualization (lead), writing – original draft (lead), writing – review and editing (equal).

## Funding

This work was supported by the Natural Science Foudation of China, 32370100; the Joint Research Program of State Key Laboratory of Agricultural and Forestry Biosecurity, SKLJRP2506; Scientific Research Project of General Administration of Customs, China, 2024HK040; Foreign Cooperation Project of Science and Technology Plan of Fujian Province, 2025I0034.

## Conflicts of Interest

The authors declare no conflicts of interest.

## Data Availability

VirPhyKit is a freely accessible online package. The source code, software package, user manual, and example data sets are available on GitHub: https://github.com/BioEasy/VirPhyKit. The VirPhyKit tutorial is available as a PDF file at https://github.com/BioEasy/VirPhyKit/master/tutorial/VirPhyKit_Tutorial.pdf.
